# Audit about Medical Decision: Data Transmission Concerning Patients with Dementia Entering French Nursing Homes Does Not Confirm the Diagnosis

**DOI:** 10.1155/2010/857581

**Published:** 2010-10-03

**Authors:** Cadier Sébastien, Hummers-Pradier Eva, Barais Marie, Barraine Pierre, Chiron Benoit, Le Floch Bernard, Nabbe Patrice, Le Reste Jean-Yves

**Affiliations:** ^1^Département de Médecine Générale, Université de Bretagne Occidentale, 29238 Brest, France; ^2^Institut für Allgemeinmedizin, Medizinische Hochschule, 30625 Hannover, Germany

## Abstract

*Background*. Dementia was affecting 855.000 patients in France in 2007. Lanmeur's rural hospital population was representative of the French nursing home's population. The followup was assumed by local GPs, which is also usual care in France for nursing homes. The study looked at clinical and paraclinical data transmitted at the institutionalization time of patients suffering from dementia. *Aim*. showing that admission letters did allow establishing a diagnosis of dementia for the GPs. *Method*. we included all patients with dementia at the time of institutionalization between July 2000 and July 2007. We searched in the admission letters for 25 criteria extracted from the French guidelines for dementia and Alzheimer disease diagnosis (multiple cross-sectional analysis per year). *Results*. 293 patients were included. The median number of diagnostic criteria present in the letters of admission is 1 (first quartile: zero, third quartile: 4, and maximum: 12). *Conclusions*. the data in admission letters did not allow the diagnosis of dementia according to the French guidelines. We know that dementia is underchecked and undertreated in France according to the same guidelines. What consequences did this lack of basic data give on motivation for treatment and recurrent diagnosis process for GPs? This has to be evaluated.

## 1. Introduction

Dementia was affecting 855. 000 patients in France in 2007 [1]. 40% of them were institutionalized [2]. ANAES (Agence nationale d'Accréditation et d'Evaluation en Santé: the National Agency of Accreditation and Evaluation in Health) guidelines since February 2000 [3], according to NINCDS-ADRDA (the National Institute of Neurological and Communicative Disorders and Stroke—Alzheimer's Disease and Related Disorders Association) had 25 criteria for positive and etiological diagnose of dementia. In France, geriatrist, psychiatrist, or neurologist did have to initiate specific treatment. Then General Practitioners (GPs) assumed followup. A specialised advice was strongly recommended in primary phase of diagnosis to have a first extensive neuropsychiatric examination. Despite large indications [4] for geriatrists, only-one third [5] of patients had specific dementia treatment in France. There is a gap between those large indications and the number of specific treatments. Could this gap come from GPs practice and on which diagnosis determinant could GPs base their practice and the medical decision to treat?

**Figure 1:**
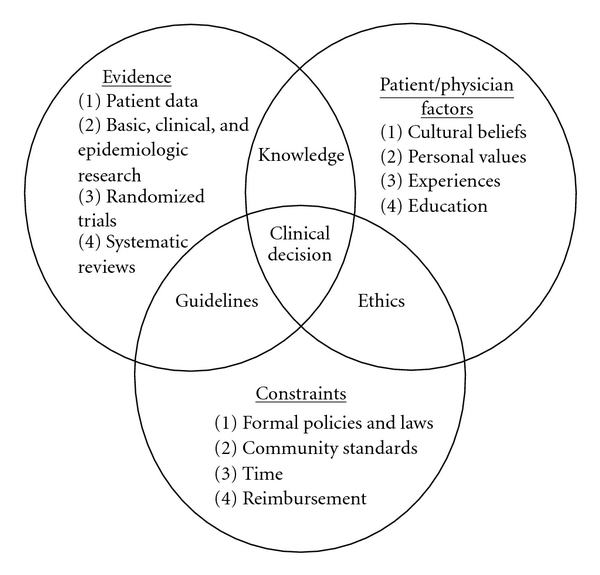
Mulrow's model.

According to Mulrow's medical decision model theory [6], medical decision is based on a tripod: Science and patient objective data, relation and communication between patient and GPs, and constraints of the health system. If we look at the decision of prescribing or not an antidementia drug, the parameters came from those three orders. In the objective data, we found different types of interrogations: is the patient suffering from Alzheimer's disease? What do we know about antidementia drugs (indications, contraindication, effectiveness, adverse drug reactions, and alternative treatments…)? In the relation data: patient's and doctor's beliefs, values, and professionalism. Among the constraints, cost, refund, political health decision (Alzheimer plan in France which push GPs to prescribe drugs) are affecting decision (see Figure 1).

For patients objective data French GPs needed clinical and paraclinical data coming from a specialized team. All those data were cost and time effective. For all those reasons, good transmission of medical data was important. We did know that generally GPs preferred data coming from the hospitalisation reports for their first input in medical records and that this attitude is emphasized by the French medical council [7]. So, the goal of our study was to show that clinical and paraclinical data in the admission letters were sufficient enough to assess the diagnosis of dementia. 

This was an important matter because most of those patients were disabled and needed medical transport for any further specialized examination. For any of those examinations, GPs were always shared between assessing a diagnosis that could change therapy and constraint (comfort) for the patient. All this has to be done in order to be both time and cost effective for a rural hospital (which, in France, is mostly a nursing home) and ethical and useful for the patients.

## 2. Method

### 2.1. Population

The studied population was living in Lanmeur's rural hospital. Care was assumed by their local GPs.

### 2.2. Place of Study

Lanmeur's rural hospital is a nursing home of 255 beds. Its population has been found representative for the population of French nursing homes according to age ratio, sex ratio, and morbidity ratio. Lanmeur has two general hospitals in the neighbourhood (Morlaix 15 km and Lannion 25 Km) and one university hospital in Brest (70 Km).

### 2.3. Inclusion

We did include all patients with coded Alzheimer disease or other dementias in ICM 10 as antecedent or diagnosis.

### 2.4. Exclusion

We did exclude patients with wrong coding (Alzheimer's plus other dementia in the same record), patients with false coding (paper charts with different diagnosis or antecedent than in computerized record), and patients with lost admission letter (letter was considered lost if it was mentioned in the medical records and not found, otherwise we supposed that we had no admission letter). Inclusion or exclusion was made from database records. Diagnosis criteria were all extracted directly and only from the original letter.

### 2.5. Search Criteria

The 25 criteria were extracted from the French national guidelines for Alzheimer's disease and dementia published by the ANAES in 2000 [3]. We did extract their attendance not their value (We did record all mentioned criteria in the entry mail neither positive or negative value) (see Table 1).

Criteria recording was done as follows for each criterio:

 Alcoholism (if found as alcoholism, alcohol, alcohol troubles),
 Familial antecedent of dementia (if mentioned),
 Vigilance state (if mentioned),
 IADL score (if quoted),
 MMS score (if quoted),
 Description of biological results: we did accept a single qualification for glycaemia (normal or not) and for serology (positive or negative) or the numbers,
 Brain imaging considered done if the result was written (for RMN or Scan) or at least mentioned.

Multicriteria as: memory impairment, cognitive tests. Cognitive testing was separated in two types: simple (5 words, clock test, verbal fluency, arithmetic test, and similarity test) and complex (Grober and Buschke test, California Verbal Learning Test, Mattis scale, and WAIS (Wechsler Adult Intelligence Scale Revised)). If at least one of those tests was written as performed the item was validated as positive.

At last, a third category of criteria needed a precise definition like evolution, neurological examination, depression, and specialized examination:

  Progressive evolution was quoted as present if the mail did mention that troubles did appear in a progressive way or if some troubles did precede a quick alteration.
 Neurological exam was quoted as present if the notion of normality of this exam was quoted or at least one description of one neurological sign.
 Depression was quoted if one result positive or negative of a test was written or if one treatment of depression was found (any type of antidepressive drugs).
 Specialized advice was quoted if it was mentioned that a geriatrist, neurologist, or psychiatrist saw the patient or if the patient was coming from such a ward.

### 2.6. Data Recording and Analysis

Every letter was taken from the files and manually read. Epi Data Entry ver 3.1 was used for recording. Epi data Analysis version 1.1 (build 68) has been used for multiple transversal analysis (on order to describe mail evolution in time). Results are in absolute value and in percentiles of patients with analysed mail (293). No significance test was used because of lack of valid valuator. Age and numbers of criteria in the mail are showed in quartile.

## 3. Results

681 patients were in our database. 378 were suffering of dementia at entry. 362 were possible to include with 30 Alzheimer's diseases and 332 dementias without aetiology. From those 362 patients, 69 were excluded because of incomplete files. This means lost letter or patient without any letter (average 23.5%, maximum 37.5%, and minimum 20% per year), wrong ICM 10 coding or different coding between manual files and electronic record (see Table 2).

From 362 patients, only 293 letters were exploitable and studied. Those patients mostly came from the three closest hospitals. They were coming from geriatric, neurologic, or internal medicine wards at almost 95% (30 different hospitalists). Medium age at entry was 83,5 years old (78,75; 88,5). Women were 206 (70,3%), and 74,5% were more than 75 years old. Six patients did arrive in 1990 or before, 11 between 1991 and 1995 to 47 in 2004, 38 in 2005, and 14 in 2006. 257 (87,71%) patients had an unidentified dementia (see Table 3).

Personal and family antecedent were written in less than 10%. Family antecedent of Alzheimer's disease was only present in one case. Mnestic troubles were described in less than 10%.

Clinical tests that could give indication for a differential diagnosis than Alzheimer's disease (evolution, associated depression, and neurological clinic anomalies) were found in, respectively, 9%, 28%, and 24%. 

Scores were very lowly present; MMS was almost the only one and was found at 11%. IADL and other specialised tests were never seen. By biological exam (especially recommended to exclude an organic aetiology of dementia) hemoglobinemia (23%), and natremia (13%) were the only ones that overrun 10%. Specialised advice was present for 43%.

The median amount of criteria present per mail is only one [0; 4] on 25 recommended by ANAES. Evolution of the median amount of criteria with time is shown in Table 4. No significative difference was seen between addressing wards or addressing practitioners.

## 4. Discussion

Here are the limits (strength and weaknesses) of our study.

 The population was representative of the French nursing homes population [8].
 Other ways of communication like phone call and electronic mail were not analyzed because, in France, they are not part of the medical records. In French real life, they were not used for medical communication and they were not liked at all by French GPs [7]. So it should not change our results.
 We had only one reviewer: it was possible to miss some data.

With those limits our study shows some surprises. 

 Diagnoses criteria of dementia written in the patient's admission letter in a French nursing home were very low. Half of those letters had only one or zero criteria on the 25 recommended by the French authorities. Even if there is a light amelioration in 2005 and 2006 (Table 4), criteria description stays very little till the end of the study and would not allow GPs confidence in diagnosis. Most of the patients were coming from the same wards for initial diagnosis, and we could incriminate those wards practice. But all of them do represent 30 different hospitalists, and our results were reproducible from one to another. It was known for long that despite the fact that the medical job should be done, communication from hospitals to GPs is not so good [9]. 

A specialised advice was done in less than half of the cases. This was a surprise because, in France, specialised advice is supposed to be compulsory to give antidementia specific drug therapy. It could be one explanation of the poor rate of drug therapy for dementia in our country. As GPs should not do the initiation of specific treatment, they have to ask for another advice in 57% of patients to do this treatment. This is an additional cost for the health system, and as it is already known that GPs are criticizing antidementia drugs effects, will they do again all the diagnosis process? 

Another surprise was that we had only 8% of alcoholism reported. The French population is a high alcohol user population and men's mortality in Brittany (our place of study) directly linked to alcoholism is very high (126.2/100000 against 70.4/100000 for all France) [10, 11]. Is this aetiology of dementia checked? And if not is the initial diagnosis in secondary care correctly done? This stays as a question.

A familial antecedent of Alzheimer's disease is an important risk factor (OR = 3,5, IC à 95%: 2,6–4,6) [12] but is only found in 1 mail on 293… is this checked too?

Sudden evolution and neurological clinical signs are for the diagnosis if Alzheimer's disease for NINCDS-ADRDA. But this is described in 9% for evolution and 24% for neurological exam. This is may be one explanation of the poor quantity of Alzheimer's disease in our study (12.29%) against 80% in the PAQUID cohort [2]. Dartigues et al. [13] showed in 2001 that 52% of patients with a specific treatment of Alzheimer's disease did had a complete initial checkup according to ANAES recommendations for clinical scores, paraclinic exams, and specialised advice. Our population is not the same with a maximum of 18% of patients under specific therapy. This gap could come from two causes: original etiological checkup are not so well done than in the PAQUID study or they are not transmitted from hospitalists.

 For the first cause it could be because recommendations are not followed. Doctors have other criteria, do not do the complete checkups or only in selectionned populations (e.g., of tip-top wards doing research as their main activity like in the PAQUID study and not only caring for people like in nursing homes). For the second one, it could be, simply, that communication between health professionals is low about dementia in France as it is low for every diagnosis between secondary and primary care [9, 14].

One last question is the impact of those incomplete letters on GPs motivation to check and treat dementia and to assume the followup of dementia patients. Mulrow's model of clinical decision [6] does insist on uncertainty as a main factor of choice for medical decision. Our admission letters with very few criteria do not help GPs with uncertainty. How does this play on dementia diagnosis and treatment in primary care in France? The literature results show only one thing: it is done for one-third of the patients (5) one explanation, between others, could come from our results enlarging uncertainty for GPs decision.

## 5. Conclusion

Despite a light amelioration since 2000 (latest publication of ANAES recommendations for followup of dementia), no complete diagnosis checkup for dementia was transmitted from secondary care to GPs in France. When it did exist (23.5% of lost or missing letters), the admission letters did not give evidence for the diagnosis of dementia. Different hypothesis could be discussed with our results: initial checkups done by hospitalist are incomplete; recommended criteria do not match with hospitalist's practice; poor medical attitude is given (herein secondary care) to vulnerable groups of the population; communication is low from hospitalists to GPs. In addition, GPs management could be criticised after being put in an awkward situation by referring colleagues. Anyway, from the patient's point of view, GPs stayed in uncertainty in front of this diagnosis without clear evidence of it. How does this play on their motivation to treat or not with specific drugs? How does this play on new checkups done at high and redundant cost for aged and disabled patients? We should go on this topic with two studies: one on GP's motivation using Mulrow's decision model with a representative GP's sample is to answer the following questions: will you keep an antidemential treatment (with the already known critics about those treatments clinical efficiency) if you have or not adverse effects while having no evidence of the trustfulness of the diagnosis? The other one on discordance between diagnosis in secondary and primary care on a representative samples of patients with dementia (according to secondary care previous diagnosis) with a new checkup done in primary care and following the French recommendations.

## Figures and Tables

**Table 1:** Criteria List.

*Antecedents*	*Paraclinic exams*
Alcoholism	TSH
Alzheimer's disease in family	Hemoglobinemia
*Illness story*	Natremia
Evolution	Calcaemia + protidemia
*Clinical exam*	Glycaemia
Vigilance	Brain imaging
Mnestic trouble	Syphilis
Depression	HIV
Neurological exam	B12
*Scores and tests*	Foliates
IADL	Hepatic Function (INR)
MMS	Lumbar Puncture
Simple cognitive test	EEG
Complex cognitive test	*Advice*
	Specialized advice

**Table 2:** Included and excluded admission letters.

Included and excluded admission letters			
	Found letter	Unfound letter	Total
Correct ICM 10 coding	293	20	313
Wrong ICM 10 coding	44	5	49

TOTAL	337	25	362

**Table 3:** Results for each recommended criteria.

Antecedents	Number (% on 293)	Paraclinic exams	Number (% on 293)
Alcoholism	8.2	TSH	7.5
Alzheimer's disease in family	0.3	Hemoglobinemia	22.9
*Illness story*		Natremia	13.3
Evolution	8.9	Calcaemia + protidemia	2.0
*Clinical exam*		Glycaemia	8.2
Vigilance	13.0	Brain imaging	24.9
Mnestic trouble	9.9	Syphilis	0.3
Depression	27.7	HIV	0.3
Neurological exam	24.2	B12	2.4
*Scores and tests*		Foliates	2.4
IADL	0.0	Hepatic Function (INR)	6.1
MMS	10.6	Lumbar Puncture	0.3
Simple cognitive test	2.4	EEG	4.1
Complex cognitive test	0.0	*Advice*	
		Specialized advice	42.7

**Table 4:** Evolution of found criteria from 1995 to 2006.

From 1995 to 1999 (*n* = 60)	2000 (*n* = 22)	2001 (*n* = 35)	2002 (*n* = 40)	2003 (*n* = 37)	2004 (*n* = 47)	2005 (*n* = 38)	2006 (*n* = 14)
1 [0.25; 4]	1 [0; 4]	1 [0; 4]	1 [0; 2]	2 [1; 4]	1 [0; 3]	3 [1; 5]	5 [1.5; 6.75]
